# Gene Expression of CD70 and CD27 Is Increased in Alopecia Areata Lesions and Associated with Disease Severity and Activity

**DOI:** 10.1155/2022/5004642

**Published:** 2022-03-08

**Authors:** Radwa El- Sayed Mahmoud Marie, Noha M. Abd El-Fadeel, Yara El-Sayed Marei, Lina M. Atef

**Affiliations:** ^1^Department of Dermatology, Venereology and Andrology, Faculty of Medicine, Suez Canal University, Ismailia, Egypt; ^2^Department of Medical Biochemistry and Molecular Biology, Faculty of Medicine, Suez Canal University, Ismailia, Egypt; ^3^Oncology Diagnostic Unit, Faculty of Medicine, Suez Canal University, Ismailia, Egypt; ^4^Department of Medical Microbiology and Immunology, Faculty of Medicine, Suez Canal University, Ismailia, Egypt

## Abstract

**Background:**

Alopecia areata (AA) is an acquired hair loss disorder induced by a cell-mediated autoimmune attack against anagen hair follicles. CD27-CD70 is a receptor-ligand complex which enhances T helper and cytotoxic T cell activation, survival, and proliferation. The overstimulation of this complex can lead to a lack of tolerance and the development of autoimmunity.

**Objectives:**

This study aimed to assess the gene expression of CD27 and CD70 in patients with AA.

**Methods:**

CD70 and CD27 mRNA expressions were evaluated by a quantitative real-time polymerase chain reaction in scalp biopsies from 40 AA patients (both AA lesions and non-lesional areas) and 40 healthy controls (HCs). The Severity of Alopecia Tool (SALT) score was used to assess AA severity. Patients were evaluated for signs of AA activity, including a positive hair pull test and dermoscopic features of black dots, broken hairs, and tapering hairs.

**Results:**

The gene expression of CD70 and CD27 was significantly higher in AA lesions than in non-lesional areas (*p* < 0.001 for both) and HCs (*p*=0.004, *p*=0.014, respectively). There were significant positive correlations between AA severity and gene expression of CD70 (*p* < 0.001) and CD27 (*p*=0.030) in AA lesions. Significant associations were detected between signs of AA activity and lesional gene expression of CD70 and CD27. Additionally, CD70 and CD27 gene expression was significantly lower in non-lesional biopsies compared to HCs (*p* < 0.001).

**Conclusion:**

Gene expression of CD70 and CD27 was increased in AA lesions and was associated with disease severity and activity. Thus, both molecules can be a predictor of AA severity and activity. Furthermore, the expression was reduced in non-lesional scalp areas. Thus, a lack of CD27 and CD70 expression may initially predispose to immunological dysregulation and the development of AA.

## 1. Introduction

Alopecia areata (AA) is an acquired non-cicatricial hair loss disorder affecting 0.1%–0.2% of individuals worldwide [1]. A collapse of the hair follicle (HF) immune privilege causing a CD8+ cytotoxic T (cT) cell assault against anagen HFs is implicated in AA development [2, 3]. However, the precise molecular mechanisms are still poorly established.

Cluster differentiation (CD) 27 is a member of the tumor necrosis factor receptor family. CD27 is expressed on natural killer cells, T helper (Th) cells, cT cells, and hematopoietic stem cells [4]. CD70, the uniquely identified ligand of CD27, belongs to the tumor necrosis factor family and is expressed exclusively on activated T cells, B cells, natural killer cells, and dendritic cells. The expression of CD70 is triggered by T cell activation upon antigen receptor attachment, toll-like receptor, or CD40 signaling. The interaction between CD70 on stimulated antigen presenting cells (APCs) and CD27 on T cells generates costimulatory signals that enhance Th and cT cell activation, survival, proliferation, chemotaxis, and cytokine production such as interleukin 2 and interferon-*γ* [5]. Moreover, CD27-CD70 interaction induces the production of CXCL10 chemokine by activated cT cells, which enhances the chemotaxis of additional activated cT cells [6]. CD70 was also found to stimulate Th1 cell differentiation independent of interleukin 12 [7]. Soluble CD27 can be shed from the activated T cells' surface via cleavage by matrix metalloproteinases to be released in the circulation. It was reported that serum soluble CD27 could enhance T cells and antigen-primed B cells' activation and increase immunoglobulin *G* production [4, 5]. Interestingly, it was proposed that CD70 signaling generated by unaroused, premature APCs can inevitably lead to a lack of tolerance and autoimmunity development [8]. Significant evidence indicates that the dysregulation of CD27-CD70 complex signaling is involved in the pathogenesis of several immune-related disorders [5], but the pathway has been poorly investigated in AA. CD70 is merely expressed upon T cell activation, and thus blocking the C27-CD70 complex has been proposed as an appealing treatment target for autoimmune disorders [9]. This study aimed to assess the expression of CD27 and CD70 genes in patients with AA and to evaluate the association between this expression and AA severity and activity.

## 2. Patients and Methods

### 2.1. Participants

A total of 40 patients with AA were enrolled in this descriptive analytical case-control study. Patients were recruited from the Dermatology Outpatient Clinic, Suez Canal University Hospital, Ismailia, Egypt. The biochemical analyses were conducted at the molecular laboratory of the Oncology Diagnostic Unit, Faculty of Medicine, Suez Canal University, Egypt. The study was performed between February and September 2021, in line with the guidelines of the Helsinki Declaration and the items of the STROBE statement. Approval was granted by the Institutional Review Board and the Research Ethics Committee, Faculty of Medicine, Suez Canal University, on 25 January 2021, with the approval code: 4454. Age and sex-matched HCs with no concurrent infections, history of AA, autoimmune disorders, atopy, or cancer were included in the study. All participants signed written informed consent form.

Alopecia areata was diagnosed clinically through detecting patches of total hair loss with normal scalp skin, and the diagnosis was confirmed by dermoscopy examination. Exclusion criteria were patients who had any systemic treatment for three months or topical applications for 2 weeks prior to the study or had regrowth of hair, concurrent infection, or a history of other autoimmune diseases or atopy or cancer.

Patients' complete history was recorded including demographic data (age and sex) and significant clinical data (duration of the present AA lesions, disease course, age of disease onset, AA in other body sites, prior attacks of AA, family history of AA, atopy, or other immune-mediated disorders), and dermatological examination was performed to detect site of patches, their number and pattern, and presence of nail abnormalities. AA clinical pattern was categorized into patchy (single patch and multiple patches), ophiasis, alopecia totalis, and alopecia universalis. The “Severity of Alopecia Tool” (SALT) score [10] was used to evaluate AA severity. AA activity was assessed via the subjective history of progression, an objective examination of the hair pull test at the edges of each patch, and the presence of black dots, broken hairs, and tapering hair on dermoscopy examination (DermLite, 3 Gen LLC, San Juan Capistrano, CA, USA) (magnification × 10).

### 2.2. Assessment of the Expression of CD70 and CD27 Genes

Punch scalp biopsies (4 mm) were taken from each patient from AA lesions and non-lesional areas. One scalp biopsy was taken from the patient with alopecia universalis and HCs. Biopsies were soon submerged in the RNA stabilizing solution (Qiagen, USA) and moved to −80 C for storage until handling. According to the manufacturer's instructions, we isolated the total RNA using RNeasy Mini Kit (QIAGEN, USA). We also assessed RNA purity by NanoDrop ND1000 spectrophotometer at the absorbance ratio of 260/280 nm (NanoDrop Tech., Inc., Wilmington, DE, USA). To assess the integrity of the RNA, we ran it at 1% agarose gel electrophoresis.

Quantitative real-time polymerase chain reactions (PCR) for CD27 and CD70 genes were performed on step one real-time PCR instrument (Applied Biosystems, UK) using COSMO cDNA synthesis kits (WF-1020500X, Willowfort, UK), HERA plus SYBR Green-qPCR Kit (WF1030800X, Willowfort, UK), and the specific primers for the target genes (Table 1).

The amplification program included two stages, an initial denaturation stage at 95  C for 3 min, followed by 40 cycles of 95 C denaturation for 15 s and annealing at 57 C for GAPDH and 60  C for (CD27 or CD70) for 60 s. After amplification, a melting curve analysis was performed to confirm PCR amplicons by collecting the fluorescence data. GAPDH was used as an internal control. The relative amounts of the target genes were calculated using the delta CT method.

### 2.3. Statistical Analysis

Statistical analysis was done via the Social Sciences Statistical Package. Categorical values were represented by using numbers and percentages. The normality of distribution was tested by the Kolmogorov–Smirnov test. Numerical values were represented using the range, median, mean, and standard deviation. Chi-square test, Monte Carlo correction, Mann–Whitney test, Kruskal–Wallis test, Wilcoxon signed rank test, and Spearman coefficient were used to measure the significance. The results were considered significant at a *p* value less than 0.05 (confidence level of 5%).

## 3. Results

### 3.1. Clinical and Demographic Data of AA Patients

In patients with AA, the age ranged from 15 to 62 years (mean age = 2 8.93 ± 12.21 years). Twenty (50%) patients were males, and 20 (50%) were females. In HCs, the age ranged from 18 to 59 (mean age = 29.80 ± 10.16 years). Eighteen (45%) HC individuals were males, and 22 (55%) were females. There was no statistically significant difference in age or gender between patients with AA and HCs (Supplementary Table 1). Clinical data of AA patients are shown in Table 2.

### 3.2. Expression of CD70 and CD27 Genes

**Figure 1:**
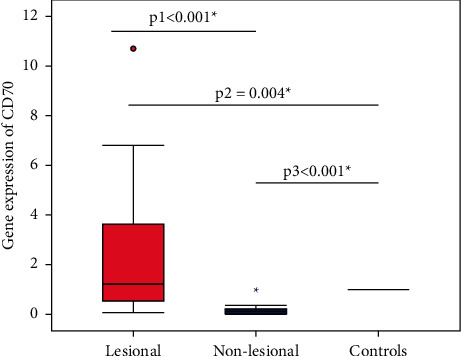
The relative expression of CD70 gene in patients with AA (*n* = 40) compared to HCs (*n* = 40). The relative CD70 gene expression in AA lesions ranged from 0.07 to 10.70 (mean 2.41 ± 2.71, median 1.22), while in non-lesional areas, it ranged from 0.01 to 0.82 (mean 0.16 ± 0.19, median 0.12). Wilcoxon signed rank test (p1) was used to compare the expression in AA lesions and non-lesional areas. Mann–Whitney test (p2) was used to compare the expression in AA lesions and HCs. Mann–Whitney test (p3) was used to compare the expression in non-lesional areas and HCs. ^∗^Significant at *p* < 0.05. AA: alopecia areata; HCs: healthy controls; CD: cluster differentiation.

The mean mRNA expression of the CD70 gene in AA lesions and non-lesional scalp skin was 2.41 fold and 0.16 fold, respectively, relative to the expression in HCs. The relative expression of CD70 gene was significantly higher in AA lesions compared to non-lesional areas (*p* < 0.001) and HCs (*p*=0.004). In addition, the gene expression was significantly lower in non-lesional areas than in HCs (*p* < 0.001) (Figure 1).

**Figure 2:**
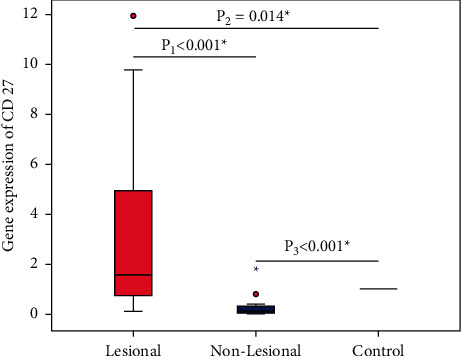
The relative expression of CD27 gene in patients with AA (*n* = 40) compared to HCs (*n* = 40). The relative CD27 gene expression in AA lesions ranged from 0.09 to 11.96 (mean 3.19 ± 3.65, median 1.58), while in non-lesional areas, it ranged from 0.0 to 1.68 (mean 0.25 ± 0.38, median 0.14). Wilcoxon signed rank test (p1) was used to compare the expression in AA lesions and non-lesional areas. Mann–Whitney test (p2) was used to compare the expression in AA lesions and HCs. Mann–Whitney test (p3) was used to compare the expression in non-lesional areas and HCs. ^∗^Significant at *p* < 0.05. AA: alopecia areata; HCs: healthy controls; CD: cluster differentiation.

The mean mRNA expression of the CD27 gene in AA lesions and non-lesional scalp skin was 3.19 fold and 0.25 fold, respectively, relative to the expression in HCs. The relative expression of the CD27 gene was significantly higher in AA lesions compared to non-lesional areas (*p* < 0.001) and HCs (*p*=0.014). The CD27 gene expression was significantly lower in non-lesional biopsies compared to HCs (*p* < 0.001) (Figure 2).

**Figure 3:**
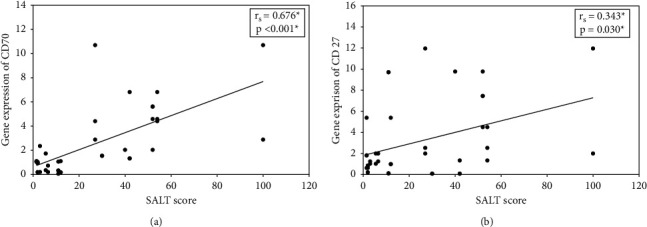
(a) The correlation between CD70 mRNA levels in AA lesions (*n* = 40) and SALT score. (b) The correlation between CD27 mRNA levels in AA lesions (*n* = 40) and SALT score. rs: Spearman coefficient; ^∗^significant at *p* < 0.05; AA: alopecia areata; SALT : Severity of Alopecia Tool; CD: cluster differentiation.

The study revealed significant positive correlations between AA severity (SALT) and the relative mRNA expressions of CD70 (*p* < 0.001) (Figure 3(a)) and CD27 (*p*=0.030) (Figure 3(b)) in AA lesions.

Furthermore, there were significant associations between signs of AA activity (positive hair pull test, and the presence of black dots, broken hairs, and tapering hair on dermoscopy examination) and the relative gene expressions of CD70 and CD27 (Table 3). Apart from that, there was no significant relation between CD70 or CD27 gene expression and other data of AA patients (age, sex, disease course, age of onset, duration of AA lesions, clinical pattern, nail abnormalities or family history of AA, atopy, or immune-mediated disorders) (Supplementary Tables 2–5).

## 4. Discussion

In this study, the relative gene expression of CD70 and CD27 in AA lesions was significantly higher than that in non-lesional areas and HCs, correlated with AA severity, and was associated with signs of AA activity, including positive hair pull test at patch margins and dermoscopic features such as black dots, broken hairs, and tapering hair. AA is generally believed to be an autoimmune assault upon anagen HF, orchestrated by Th1 and cT lymphocytes. Inflammatory infiltrate of Th, cT cells, and APCs was identified in the peribulbar region of anagen HFs in active AA lesions and was found to be correlated with AA severity. These kinds of infiltrate trigger apoptosis in anagen HF keratinocytes, causing their arrest and regression into the telogen or dystrophic anagen states [2]. Clinically, this is manifested by a sudden stoppage of hair shaft growth, resulting in tapered and broken hair shafts with a positive hair pull test at the active patch margin [11]. The CD27-CD70 complex is mostly expressed on activated T cells, B cells, natural killer cells, and dendritic cells, and its interaction signaling facilitates Th1 and cT cell activation, survival, proliferation, and chemotaxis [5]. Thus, the higher gene expression of CD70 and CD27 in active AA lesions can be explained by the increased infiltration of immune cells expressing these molecules. This infiltration was present mostly in active AA lesions and was associated with AA severity; this fact may clarify the association between CD27 and CD70 gene expression and AA severity and signs of activity. As a result, CD27 and CD70 gene expression can be used to predict the severity and activity of AA. To date, no previous studies have evaluated the gene expression of CD27-CD70 complex in AA.

In agreement with our results, CD70 expression was upregulated on CD4+ Th cells isolated from the synovial fluid of patients with psoriatic arthritis [12]. CD70 expression was also increased in the synovium and peripheral blood mononuclear cells (PBMCs) of patients with rheumatoid arthritis [12, 13], systemic lupus erythematosus (SLE) [14], systemic sclerosis [15], and Sjogren's syndrome [16]. This expression correlated with the hypo or demethylation of the CD70 gene promoter area, which causes a failure to repress CD70 expression when it is triggered by T cell activation [15, 16]. Regarding CD27, its soluble serum level was elevated in patients with active vitiligo and was suggested as a marker of disease progression [17, 18]. Serum soluble CD27 was downregulated upon treatment of psoriasis [19]. CD27 expression was significantly elevated in the lesional skin and serum of patients with systemic sclerosis, with a significant association with disease severity [20]. The expression of CD27+ B cells and serum soluble CD27 was increased in SLE patients and correlated with disease activity [21]. The pathogenesis of most of these diseases entails cell-mediated autoimmune inflammatory pathways, and their association with AA is well established [2]. In the same manner, the expression of CD70 was increased in human contact dermatitis, which is a Th1-mediated inflammation [22].

Additionally, the present study revealed that CD27 and CD70 gene expression was significantly lower in the non-lesional areas compared to HCs. Interestingly, Abolhassani [23] reported two family members with genetic abnormalities in the CD70- CD27 signaling cascade associated with clinical features of AA, Behcet's disease, recurrent viral pneumonia, central nervous system infection, and Hodgkin lymphoma induced by Epstein–Barr virus. The patients' clinical and immunologic data revealed an abnormality in B-cell differentiation, impaired functional activity of effector T cells, and decreased antibody production, which increased vulnerability to recurrent viral illness. The authors proposed an association between CD70 deficiency and an increased risk of alopecia areata due to either recurrent uncontrolled viral infections or decreased proliferation and activity of T-regulatory cells. This study's findings are consistent with our results of decreased CD70 and CD27 gene expression in the non-lesional scalp areas of AA patients, suggesting that a deficiency of CD70 and CD27 expression may predispose to immunological dysregulation and the development of AA. After that, the recruitment of autoreactive T cells against anagen hair follicles in active AA lesions may cause local overexpression of CD70 and CD27 expressed on the infiltrating immune cells.

Notably, several in vivo studies have suggested that monoclonal antibodies targeting CD27-CD70 complex could be a potential therapeutic modality in autoimmune diseases [5]. Anti-CD70 antibodies lowered the antibody titer and decreased joint disease's severity in murine collagen-induced arthritis [9]. In addition, anti-CD70 antibodies repressed immunoglobulin secretion by B cells triggered by T cells isolated from SLE patients [24]. Colitis was prevented along with a decrease in colitis-associated Th1 cytokines in a mouse model using anti-CD70 antibodies [25]. Accordingly, our study findings may shed new light on targeting CD27-CD70 complex for medical treatment of AA, especially severe cases resistant to traditional medical treatment.

Indeed, the conclusions of this study should be considered against its limitations, which include small sample size and the lack of evaluation of CD27 and CD70 tissue expression in AA scalp lesions compared to non-lesional areas and HCs. Moreover, additional research investigating the molecular functions of CD27 and CD70 in AA and comparing the expression of both molecules during AA activity and after recovery and hair regrowth is needed.

## 5. Conclusion

The expression of CD27 and CD70 genes was increased in AA scalp lesions and was associated with AA severity and activity. CD27-CD70 interaction can therefore be a predictor of AA severity and activity. Furthermore, the expression of both molecules was lower in non-lesional scalp areas. Thus, a lack of CD27 and CD70 expression may predispose to immunological dysregulation and the development of AA.

## Figures and Tables

**Table 1:** Primers used for real-time polymerase reaction assay.

Gene	Sequence
CD70	Forward: 5′- TTGGTGATCTGCCTCGTGGT-3′
	Reverse: 5′- CCTTGTCCAGCTCTGGTCCAT-3′
CD27	Forward: 5′- ACCCTCAGCCCACCCACTTA-3′
	Reverse: 5′- CAGGGTGAAAACAAGGAACATT-3′
GAPDH	Forward: 5′-CGGAGTCAACGGATTTGGTCGTAT- 3′
	Reverse: 5′-AGCCTTCTCCATGGTGGT-3′

CD: cluster differentiation; GAPDH : human glyceraldehyde-3-phosphate dehydrogenase.

**Table 2:** Clinical data of patients with AA (*n* = 44).

Clinical data	No.	%
Duration of AA lesions (months)		
<3 months	8	20.0
3–12 months	11	27.5
12–24 months	5	12.5
2–5 years	9	22.5
≥5 years	7	17.5
Min.–Max.	0.25–168.0	
Mean ± SD	26.98 ± 39.75	
Median (IQR)	12.0 (3.0–36.0)	
Course		
Progressive	27	67.5
Fluctuating	5	12.5
Stationary	8	20.0
Age of disease onset (years)		
Children (0- <13)	6	15.0
Adolescents and young youth (13- <25)	16	40.0
Adulthood (25- <40)	10	25.0
Middle age (40- <60)	8	20.0
Min.–Max.	2.0–50.0	
Mean ± SD	25.58 ± 13.25	
Median (IQR)	24.0 (17.0–32.0)	
Alopecia in other sites (beard or eyebrows)	14	35.0
SALT score		
S1 (<25% hair loss)	22	55.0
S2 (25–49% hair loss)	9	22.5
S3 (50–74% hair loss)	7	17.5
S4 (75–99% hair loss)	0	0.0
S5 (100% hair loss)	2	5.0
Min.–Max.	1.50–100.0	
Mean ± SD	25.08 ± 26.02	
Median (IQR)	12.0 (3.0–42.0)	
Pattern		
Patchy	22	55.0
Single patch	7	17.5
Multiple separate patches	12	30.0
Multiple reticular patches	3	7.5
Ophiasis ± other patterns	16	40.0
Ophiasis	8	20.0
Multiple separate patches and ophiasis	7	17.5
Ophiasis and sisaipho	1	2.5
Universalis	2	5.0
Previous attacks of AA	22	55.0
Family history of AA	3	7.5
Family history of atopy	5	12.5
Family history of autoimmune disorders	3	7.5
Nail changes (pitting, ridging, dystrophy)	7	17.5
Black dots	30	75.0
Broken hairs	16	40.0
Tapering hairs	26	65.0
Positive hair pull test	22	55.0

SD: standard deviation; IQR: interquartile range. AA: alopecia areata; SALT : Severity of Alopecia Tool.

**Table 3:** Relation between CD70 and CD27 gene expression in AA lesions and signs of AA activity (positive hair pull test and dermoscopic features) (*n* = 40).

	N	CD 70 in AA lesion			Test of sig.	*p*	CD27 in AA lesion			Test of sig.	*P*
		Mean ± SD	Median	IQR (25th–75th)			Mean ± SD	Median	IQR (25th–75th)		
Black dots											
Absent	10	0.69 ± 0.59	0.57	1.01 (0.18–1.07)	*U* = 48.0 ^*∗*^	0.001 ^*∗*^	0.45 ± 0.41	0.21	0.78 (0.12–0.85)	*U* = 24.0^*∗*^	<0.001 ^*∗*^
Present	30	2.98 ± 2.90	1.89	3.56 (1.04–4.59)			4.10 ± 3.79	2.0	6.22 (1.25–7.46)		
Broken hair											
Absent	24	1.65 ± 2.86	1.04	1.28 (0.21–1.44)	*U* = 68.0 ^*∗*^	<0.001 ^*∗*^	1.71 ± 2.56	1.01	1.71 (0.37–1.91)	*U* = 68.0 ^*∗*^	<0.001 ^*∗*^
Present	16	3.55 ± 2.07	3.65	4.02 (1.58–5.11)			5.40 ± 3.98	4.94	7.57 (1.93–8.62)		
Tapering hair											
Absent	14	0.81 ± 0.64	0.95	0.93 (0.21–1.07)	*U* = 69.0 ^*∗*^	0.001 ^*∗*^	0.60 ± 0.45	0.63	0.91 (0.12–1.03)	*U* = 36.0 ^*∗*^	<0.001 ^*∗*^
Present	26	3.27 ± 3.01	2.04	3.76 (1.09–4.59)			4.58 ± 3.85	2.53	6.33 (1.82–7.46)		
Positive hair pull test											
Absent	18	0.82 ± 0.65	0.95	1.13 (0.20–1.33)	*U* = 55.0 ^*∗*^	<0.001 ^*∗*^	0.58 ± 0.45	0.63	0.89 (0.12–0.99)	*U* = 0.0 ^*∗*^	<0.001 ^*∗*^
Present	22	3.71 ± 3.06	2.89	4.51 (1.11–5.62)			5.32 ± 3.74	4.50	7.71 (2.0–9.71)		

SD: standard deviation; IQR: interquartile range. U : Mann–Whitney test. ^∗^Significant at *p* value less than 0.05. AA: alopecia areata; CD: cluster differentiation.

## Data Availability

The data and materials related to the present work are included within this article.

## Abbreviations

AA: Alopecia areata
HF: Hair follicle
cT: Cytotoxic *T*
CD: Cluster differentiation
Th: T helper
APCs: Antigen presenting cells
HCs: Healthy controls
SALT: Severity of Alopecia Tool
PCR: Polymerase chain reactions
GAPDH: Glyceraldehyde-3-phosphate dehydrogenase
PBMNCs: Peripheral blood mononuclear cells
SLE: Systemic lupus erythematosus.

## Supplementary Materials

Supplementary Materials Supplementary table 1: demographics of the study participants (*n* = 80). Supplementary table 2: relation between CD70 gene expression in AA lesions and different data of patients with AA (*n* = 40). Supplementary table 3: correlation between CD70 gene expression in AA lesions and different data of patients with AA (*n* = 40). Supplementary table 4: relation between CD27 gene expression in AA lesions and different data of patients with AA (*n* = 40). Supplementary table 5: correlation between CD27 gene expression in AA lesions and different data of patients with AA (*n* = 40).
