# Supplementary material for: Gene Expression of CD70 and CD27 Is Increased in Alopecia Areata Lesions and Associated with Disease Severity and Activity
Source: Dermatol Res Pract. 2022 Mar 8;2022:5004642. doi: 10.1155/2022/5004642 (PMC8923777; doi:10.1155/2022/5004642)
Supplement: Supplementary Materials — Supplementary table 1: demographics of the study participants (n = 80). Supplementary table 2: relation between CD70 gene expression in AA lesions and different data of patients with AA (n = 40). Supplementary table 3: correlation between CD70 gene expression in AA lesions and different data of patients with AA (n = 40). Supplementary table 4: relation between CD27 gene expression in AA lesions and different data of patients with AA (n = 40). Supplementary table 5: correlation between CD27 gene expression in AA lesions and different data of patients with AA (n = 40). [file 5004642.f1.zip › 5004642.f1/Supplementary table 1.docx]

**Supplementary table 1:** Demographics of patients with AA (n= 40), and HCs (n= 40).

|  | **Patients with AA (n = 40)** | | **HCs (n = 40)** | | **Test of Sig.** | ***P* value** |
| --- | --- | --- | --- | --- | --- | --- |
|  | **No.** | **%** | **No.** | **%** |  |  |
| **Gender** |  |  |  |  |  |  |
| Male | 20 | 50.0 | 18 | 45.0 | χ^2^= 0.201 | 0.654 |
| Female | 20 | 50.0 | 22 | 55.0 |  |  |
| **Age (years)** |  |  |  |  |  |  |
| Adolescents & young youth (13- <25) | 18 | 45.0 | 18 | 45.0 | χ^2^= 0.186 | ^MC^p= 1.000 |
| Adulthood (25- <40) | 13 | 32.5 | 13 | 32.5 |  |  |
| Middle age (40- <60) | 7 | 17.5 | 7 | 17.5 |  |  |
| Old age (≥ 60) | 2 | 5.0 | 2 | 005.0 |  |  |
| Min. – Max. | 15.0 – 62.0 | | 15.0 – 62.0 | | U= 796.50 | 0.973 |
| Mean ± SD. | 28.93 ± 12.21 | | 28.80 ± 12.16 | |  |  |
| Median (IQR) | 28.0(20.0 – 33.50) | | 25.50(20.0 – 32.5) | |  |  |

χ^2^: Chi square test; U: Mann Whitney test; SD: Standard deviation; AA: Alopecia areata. Significant *p* value is less than 0.05.
