# Supplementary material for: Gene Expression of CD70 and CD27 Is Increased in Alopecia Areata Lesions and Associated with Disease Severity and Activity
Source: Dermatol Res Pract. 2022 Mar 8;2022:5004642. doi: 10.1155/2022/5004642 (PMC8923777; doi:10.1155/2022/5004642)
Supplement: Supplementary Materials — Supplementary table 1: demographics of the study participants (n = 80). Supplementary table 2: relation between CD70 gene expression in AA lesions and different data of patients with AA (n = 40). Supplementary table 3: correlation between CD70 gene expression in AA lesions and different data of patients with AA (n = 40). Supplementary table 4: relation between CD27 gene expression in AA lesions and different data of patients with AA (n = 40). Supplementary table 5: correlation between CD27 gene expression in AA lesions and different data of patients with AA (n = 40). [file 5004642.f1.zip › 5004642.f1/supplementary table 2.docx]

**Supplementary table 2:** Relation between CD70 gene expression in AA lesions and the different data of patients with AA (n= 40).

|  | **N** | **CD 70 in AA lesions** | | | **Test of Sig.** | **p** |
| --- | --- | --- | --- | --- | --- | --- |
|  |  | **Mean ± SD.** | **Median** | **IQR (25th – 75th)** |  |  |
| **Gender** |  |  |  |  |  |  |
| Male | **20** | 2.88 ± 3.19 | 1.44 | 3.45 (0.95 – 4.41) | U= 166.0 | 0.369 |
| Female | **20** | 1.94 ± 2.11 | 1.08 | 1.94 (0.35 – 2.20) |  |  |
| **Course** |  |  |  |  |  |  |
| Progressive | **27** | 1.86 ± 1.83 | 1.11 | 1.62 (0.85 – 2.20) | H= 0.861 | 0.650 |
| Fluctuating | **5** | 4.58 ± 5.60 | 1.09 | 10.51 (0.20 – 10.70) |  |  |
| Stationary | **8** | 2.91 ± 2.41 | 2.96 | 4.93 (0.65 – 5.11) |  |  |
| **Alopecia in other sites** |  |  |  |  | U= 163.0 | 0.604 |
| No | **26** | 2.38 ± 2.95 | 1.21 | 2.59 (0.36 – 2.36) |  |  |
| Yes | **14** | 2.48 ± 2.31 | 1.22 | 3.55 (1.04 – 4.41) |  |  |
| **Pattern** |  |  |  |  | H= 3.602 | 0.165 |
| Patchy | **22** | 3.41 ± 3.28 | 2.04 | 4.82 (0.95 – 5.62) |  |  |
| Ophiasis & other | **16** | 1.22 ± 0.92 | 1.11 | 1.36 (0.35 – 1.64) |  |  |
| Universalis | **2** | 1.04 ± 0.0 | 1.04 | – (–) |  |  |
| **Previous episode** |  |  |  |  | U= 172.0 | 0.492 |
| No | **18** | 1.90 ± 1.90 | 1.10 | 2.59 (0.36 – 2.36) |  |  |
| Yes | **22** | 2.83 ± 3.22 | 1.44 | 3.77 (0.74 – 4.41) |  |  |
| **Nail changes** |  |  |  |  |  |  |
| No | **33** | 2.62 ± 2.92 | 1.33 | 4.16 (0.36 – 4.41) | U= 87.50 | 0.326 |
| Yes | **7** | 1.42 ± 1.05 | 1.04 | 1.94 (0.95 – 1.96 ) |  |  |
| **Family history** |  |  |  |  | H= 4.100 | 0.251 |
| Negative | **29** | 2.13 ± 2.17 | 1.11 | 4.08 (0.33 – 4.41) |  |  |
| Alopecia | **3** | 7.58 ± 5.41 | 10.70 | – (6.02 – 10.70) |  |  |
| Atopy | **5** | 1.60 ± 1.22 | 1.04 | 2.28 (1.04 – 2.89) |  |  |
| Autoimmune | **3** | 1.27 ± 0.50 | 1.33 | – (1.04 – 1.53) |  |  |

**U:** Mann Whitney test; H: Kruskal Wallis test; *: Statistically significant at p< 0.05. SD: standard deviation; IQR: interquartile range; AA: alopecia areata; CD: cluster differentiation.
