# Supplementary material for: Gene Expression of CD70 and CD27 Is Increased in Alopecia Areata Lesions and Associated with Disease Severity and Activity
Source: Dermatol Res Pract. 2022 Mar 8;2022:5004642. doi: 10.1155/2022/5004642 (PMC8923777; doi:10.1155/2022/5004642)
Supplement: Supplementary Materials — Supplementary table 1: demographics of the study participants (n = 80). Supplementary table 2: relation between CD70 gene expression in AA lesions and different data of patients with AA (n = 40). Supplementary table 3: correlation between CD70 gene expression in AA lesions and different data of patients with AA (n = 40). Supplementary table 4: relation between CD27 gene expression in AA lesions and different data of patients with AA (n = 40). Supplementary table 5: correlation between CD27 gene expression in AA lesions and different data of patients with AA (n = 40). [file 5004642.f1.zip › 5004642.f1/Supplementary table 3.docx]

**Supplementary table 3:** Correlation between CD70 gene expression in AA lesions and the different data of patients with AA (n= 40).

|  | **CD 70 in AA lesions** | |
| --- | --- | --- |
|  | **r_s_** | **p** |
| **Age (years)** | -0.154 | 0.344 |
| **Duration of AA lesions (months)** | 0.033 | 0.839 |
| **Age of disease onset (years)** | -0.200 | 0.216 |
| **SALT score** | 0.676 | <0.001^*^ |

r_s_: Spearman coefficient. *: Statistically significant at p < 0.05. AA: alopecia areata; CD: cluster differentiation.
