# Supplementary material for: Gene Expression of CD70 and CD27 Is Increased in Alopecia Areata Lesions and Associated with Disease Severity and Activity
Source: Dermatol Res Pract. 2022 Mar 8;2022:5004642. doi: 10.1155/2022/5004642 (PMC8923777; doi:10.1155/2022/5004642)
Supplement: Supplementary Materials — Supplementary table 1: demographics of the study participants (n = 80). Supplementary table 2: relation between CD70 gene expression in AA lesions and different data of patients with AA (n = 40). Supplementary table 3: correlation between CD70 gene expression in AA lesions and different data of patients with AA (n = 40). Supplementary table 4: relation between CD27 gene expression in AA lesions and different data of patients with AA (n = 40). Supplementary table 5: correlation between CD27 gene expression in AA lesions and different data of patients with AA (n = 40). [file 5004642.f1.zip › 5004642.f1/Supplementary table 4.docx]

**Supplementary table 4:** Relation between CD27 gene expression in AA lesions and the different data of patients with AA (n= 40).

|  | **N** | **CD27 in AA lesion skin** | | | **Test of Sig.** | **p** |
| --- | --- | --- | --- | --- | --- | --- |
|  |  | **Mean ± SD.** | **Median** | **IQR (25th – 75th)** |  |  |
| **Gender** |  |  |  |  |  |  |
| Male | **20** | 3.27 ± 3.77 | 1.91 | 5.02 (0.53 – 5.39) | U= 196.0 | 0.369 |
| Female | **20** | 3.11 ± 3.61 | 1.34 | 3.78 (0.81 – 4.50) |  |  |
| **Course** |  |  |  |  |  |  |
| Progressive | **27** | 3.39 ± 4.13 | 1.34 | 5.18 (0.42 – 5.39) | H= 0.609 | 0.738 |
| Fluctuating | **5** | 1.50 ± 0.60 | 1.82 | 1.15 (0.85 – 2.0) |  |  |
| Stationary | **8** | 3.54 ± 2.88 | 3.16 | 5.47 (1.25 – 5.98) |  |  |
| **AA in other sites** |  |  |  |  | U= 126.0 | 0.116 |
| No | **26** | 2.43 ± 2.96 | 1.25 | 2.42 (0.21 – 2.0) |  |  |
| Yes | **14** | 4.59 ± 4.44 | 2.53 | 8.81 (0.99 – 9.71) |  |  |
| **Pattern** |  |  |  |  | H= 2.854 | 0.240 |
| Patchy | **22** | 3.33 ± 3.30 | 1.91 | 4.09 (1.25 – 4.50) |  |  |
| Ophiasis & other | **16** | 3.31 ± 4.28 | 1.03 | 5.27 (0.12 – 5.39) |  |  |
| Universalis | **2** | 0.63 ± 0.0 | 0.63 | – (–) |  |  |
| **Previous episode** |  |  |  |  | U= 176.0 | 0.563 |
| No | **18** | 3.46 ± 3.39 | 1.53 | 5.11 (0.99 – 5.39) |  |  |
| Yes | **22** | 2.96 ± 3.91 | 1.58 | 1.90 (0.63 – 2.53) |  |  |
| **Nail changes** |  |  |  |  |  |  |
| No | **33** | 3.06 ± 3.20 | 1.82 | 4.02 (0.99 – 4.50) | U=  98.50 | 0.553 |
| Yes | **7** | 3.80 ± 5.58 | 0.63 | 11.75 (0.42 – 6.48) |  |  |
| **Family history** |  |  |  |  |  |  |
| Negative | **29** | 3.21 ± 3.39 | 1.34 | 4.54 (0.85 – 5.39) | H= 0.536 | 0.911 |
| Alopecia | **3** | 1.36 ± 1.10 | 2.0 | – (1.05 – 2.0) |  |  |
| Atopy | **5** | 5.24 ± 6.14 | 0.99 | 11.33 (0.63 – 11.96) |  |  |
| Autoimmune | **3** | 1.36 ± 1.10 | 2.0 | – (1.05 – 2.0) |  |  |

**U:** Mann Whitney test; H: Kruskal Wallis test; *: Statistically significant at p< 0.05. SD: standard deviation; IQR: interquartile range; AA: alopecia areata; CD: cluster differentiation.
